# Comparable efficacy with similarly low risk of hypoglycaemia in patient‐ vs physician‐managed basal insulin initiation and titration in insulin‐naïve type 2 diabetic subjects: The Italian Titration Approach Study

**DOI:** 10.1002/dmrr.3304

**Published:** 2020-04-05

**Authors:** Riccardo C. Bonadonna, Andrea Giaccari, Raffaella Buzzetti, Gianluca Perseghin, Domenico Cucinotta, Angelo Avogaro, Gianluca Aimaretti, Monica Larosa, Carmine G. Fanelli, Geremia B. Bolli

**Affiliations:** ^1^ Division of Endocrinology and Metabolic Diseases and Department of Medicine and Surgery University of Parma and AOU of Parma Italy Parma Italy; ^2^ Fondazione Policlinico Universitario A. Gemelli IRCCS, Rome and Università Cattolica del Sacro Cuore Rome Italy; ^3^ Sapienza University of Rome Rome Italy; ^4^ University of Milan Bicocca Milan Italy; ^5^ University of Messina Messina Italy; ^6^ University of Padua Padova Italy; ^7^ University of the Eastern Piedmont Vercelli Italy; ^8^ Sanofi Milan Italy; ^9^ Section of Endocrinology and Metabolism, Department of Medicine Perugia University Medical School Perugia Italy

**Keywords:** glargine 300 U/mL, glycaemic control, hypoglycaemia, insulin analogues, personalized medicine, randomized trial, type 2 diabetes

## Abstract

**Aims:**

People with uncontrolled type 2 diabetes (T2DM) often delay initiating and titrating basal insulin. Patient‐managed titration may reduce such deferral. The Italian Titration Approach Study (ITAS) compared the efficacy and safety of insulin glargine 300 U/mL (Gla‐300) initiation and titration using patient‐ (nurse‐supported) or physician‐management in insulin‐naïve patients with uncontrolled T2DM.

**Materials and methods:**

ITAS was a multicentre, phase IV, 24‐week, open‐label, randomized (1:1), parallel‐group study. Insulin‐naïve adults with T2DM for ≥1 year with poor metabolic control initiated Gla‐300 after discontinuation of SU/glinides, and were randomized to self‐titrate insulin dose (nurse‐assisted) or have it done by the physician. The primary endpoint was change in HbA_1c_. Secondary outcomes included hypoglycaemia incidence and rate, change in fasting self‐monitored plasma glucose, patient‐reported outcomes (PROs), and adverse events.

**Results:**

Three hundred and fifty five participants were included in the intention‐to‐treat population. At Week 24, HbA_1c_ reduction from baseline was non‐inferior in patient‐ vs physician‐managed arms [least squares mean (LSM) change (SE): −1.60% (0.06) vs −1.49% (0.06), respectively; LSM difference: −0.11% (95% CI: −0.26 to 0.04)]. The incidence and rates of hypoglycaemia were similarly low in both arms: relative risk of confirmed and/or severe nocturnal (00:00‐05:59 hours) hypoglycaemia was 0.77 (95% CI: 0.27 to 2.18). No differences were observed for improvement in PROs. No safety concerns were reported.

**Conclusions:**

In the T2DM insulin‐naïve, SU/glinides discontinued population, patient‐managed (nurse‐assisted) titration of Gla‐300 may be a suitable option as it provides improved glycaemic control with low risk of hypoglycaemia, similar to physician‐managed titration.

## 1 INTRODUCTION

There is often a delay in initiation and titration of basal insulin (BI) in people with type 2 diabetes mellitus (T2DM) inadequately controlled by non‐insulin treatment.^1^, ^2^ Lack of time and resources for healthcare providers (HCPs), complexity of the titration process, fear of hypoglycaemia,^3^, ^4^, ^5^ along with psychological barriers to the idea itself of insulin initiation, are the most common reasons of such a delay.

To overcome these barriers, physicians recommend educational tools and support from medical staff.^4^ Indeed, nurse‐assisted insulin treatment has been shown to improve initiation rates and provide greater HbA_1c_ reductions compared with usual care.^6^, ^7^ Simple titration algorithms have also been shown to help patients to self‐manage their titration.^8^ Results from the AT.LANTUS and ATLAS studies, which compared patient‐ and physician‐managed titration of glargine 100 U/mL (Gla‐100), showed that patient‐managed titration provided slightly, albeit significantly, better HbA_1c_ reductions and was well tolerated.^8^, ^9^

Insulin glargine 300 U/mL (Gla‐300) has more stable pharmacokinetic and pharmacodynamic profiles compared with Gla‐100 at fixed^10^ and, most importantly, at clinical doses used by patients with type 1 diabetes (T1DM),^11^, ^12^ and reduces the risk of hypoglycaemia vs Gla‐100 in T2DM.^13^ Thus, self‐titration with Gla‐300 might offer advantages to patients to effectively and safely control hyperglycaemia. A recent study (TAKE CONTROL^14^) has compared patient‐ vs physician‐managed titration of Gla‐300. However, this comparison was evaluated in a T2DM population heterogeneous for antecedent insulin use and continued use of sulphonylureas (SU). No study has so far examined the question specifically in a homogeneous group of insulin‐naïve T2DM people in the absence of SU or glinides.

The Italian Titration Approach Study (ITAS) aimed to test the efficacy and safety of initiation and titration of Gla‐300 with the same algorithm used either by patients (nurse‐assisted) or by physicians in people with uncontrolled T2DM naïve to insulin.

## 2 MATERIALS AND METHODS

### 2.1 Trial design and participants

ITAS was a national, multicentre, phase IV, 24‐week, open‐label, randomized (1:1), parallel‐group study, conducted in Italy (EudraCT Number: 2015‐001167‐39). The primary aim of this study was to assess non‐inferiority of change in HbA_1c_ over 24 weeks, when the same algorithm for Gla‐300 dose titration was managed by the patient (nurse‐assisted) or the physician. Participants were insulin‐naïve adults (≥18 years of age) with T2DM for ≥1 year with poor glycaemic control (HbA_1c_ ≥7.5 to ≤10%) on oral antihyperglycaemic drugs (OADs) and/or non‐insulin injectables. After withdrawal of SU/glinides, if any, to evaluate the hypoglycaemia risk solely due to Gla‐300, all participants were treated with Gla‐300 and were randomized to either self‐adjust their BI dose, or have their dose adjusted by a physician during visits or by telephone. The study consisted of a 2‐week screening period and a 24‐week treatment period (with interim analysis at 12 weeks). In each arm, visits/contacts were weekly until week 12, and then every 2 weeks until week 24. Additional unscheduled contacts (phone, on‐site visit) were made available if clinically required. Full details of the methodology have recently been reported.^15^

All participants provided informed, written consent. The clinical trial protocol was approved by the appropriate local Ethical Committees and IRB/IEC. The study was conducted in accordance with the Declaration of Helsinki and the ICH guidelines for good clinical practice.^16^

### 2.2 Basal insulin titration

All patients were instructed to self‐administer a daily subcutaneous injection of Gla‐300 (Toujeo SoloStar®, Sanofi) in the evening, anytime from dinner to bedtime. Gla‐300 was administered at a starting dose of 0.2 U/kg and then adjusted at each visit in the physician‐managed arm, and weekly or even more frequently (but no more often than every 3‐4 days) in the patient (nurse‐assisted) managed arm to achieve a fasting self‐monitored plasma glucose (SMPG) of 80‐110 mg/dL. In both groups, changes in the insulin dose were based on the median of the fasting SMPG values measured on three consecutive days, of which the last was the day when titration was scheduled. Patients randomized to self‐managing insulin titration received a specific educational session regarding self‐adjustment of insulin dose from the study nurse, who monitored algorithm application without however exerting any influence on titration. The patient‐ and physician‐managed titration algorithm is shown in Table S1.

Rescue therapy, if needed, either by adding a new OAD and/or by increasing the dose of an existing antihyperglycaemic non‐study drug, was based on the Investigator's judgement, considering primarily the patient's individual clinical needs, but also local guidelines and Gla‐300 labelling.^17^ Further details of the titration protocol are also provided in the Appendix.

At randomization, investigators provided patients with a blood glucometer (MyStar Extra®; Sanofi) and diary to assess and record daily fasting SMPG until it was stable at the target. A seven‐point SMPG profile was measured at Week 12 and Week 24. The investigator explained the need to measure glucose at the times requested by the study and to record the values correctly in the diary that the patients brought along with the glucometer at each office visit.

Patients were instructed to measure capillary plasma glucose whenever they experienced symptoms of hypoglycaemia. All hypoglycaemia episodes were recorded in the patient's diary or documented in the “hypoglycaemia screen/page” of the electronic case report form (e‐CRF). Nocturnal hypoglycaemia was any event that occurred between 00:00 and 05:59. Severe hypoglycaemia was defined as an event requiring assistance of another person to actively administer carbohydrate or glucagon or to perform other resuscitative actions.

### 2.3 Endpoints and other assessments

The primary endpoint of the study was change in HbA_1c_ from baseline to Week 24 (assessed using 0.3% as non‐inferiority margin). The main secondary endpoint was the percentage of participants with ≥1 confirmed [≤70 mg/dL (≤3.9 mmol/L)] and/or severe nocturnal (00:00‐05:59 hours) hypoglycaemic event from baseline to Week 24. Other efficacy endpoints included percentage of participants with ≥1 confirmed [≤70 mg/dL (≤3.9 mmol/L)] and/or severe hypoglycaemic event during any time of day (24 hours) or between 00:00 hour and pre‐breakfast (an expanded nocturnal window), and the number of hypoglycaemic events per patient‐year during the study treatment; the cut‐off <54 mg/dL was also investigated for each category of hypoglycaemia. Other secondary endpoints included change from baseline to Week 24 in fasting plasma glucose (FPG), fasting SMPG (a seven‐point SMPG profile was performed at Week 12 and Week 24), insulin dose, body weight, and percentage of patients with HbA_1c_ target achievement (<7.0%/<7.5%/<8.0%; without severe and/or confirmed hypoglycaemia and without severe and/or confirmed hypoglycaemia and weight gain at Weeks 12 and 24).

Other assessments included patient‐reported outcomes (PROs), which were collected using the following: Problem Areas in Diabetes‐5 (PAID‐5) questionnaire, Diabetes Empowerment Scale – short form (DES‐SF) questionnaire, and Diabetes Treatment Satisfaction Questionnaire (DTSQ). Safety and tolerability analyses were based on all hypoglycaemic events, skin reaction at injection site, hypersensitivity reactions, and any other adverse events (AE) or serious adverse events (SAE).

### 2.4 Statistical analyses

Full details of the statistical analyses have previously been reported.^15^ The primary efficacy analysis was performed in the intention‐to‐treat (ITT) population, comprising all randomized patients who received at least one dose of Gla‐300 and had a baseline assessment of primary efficacy variables, irrespective of compliance with the study protocol and procedures. The per‐protocol (PP) population consisted of all patients in the ITT population without major protocol deviations and was used for the supportive analysis of the primary efficacy endpoint. The safety population was defined as all randomized patients who received at least one dose of Gla‐300 and had at least one safety variable collected.

The primary endpoint was analysed using a linear mixed‐effect model (LMEM) with titration approach and centre as the fixed effect and the HbA_1c_ baseline value as the covariate. To assess the non‐inferiority of patient‐managed vs physician‐managed titration, the upper bound of the 95% confidence interval (CI) for the estimated difference in the mean change of HbA_1c_ from baseline to endpoint at Week 24 between the two titration approaches was compared with the predefined non‐inferiority margin of 0.3% HbA_1c_. Non‐inferiority was shown if the upper bound of the two‐sided 95% CI of the estimated difference for the ITT population was <0.3%.

The risk of hypoglycaemic events in the two titration groups was compared in terms of relative risk, whereas a rate ratio was computed to compare the annual incidence rates of same type of events in the two‐titration arms. All statistical comparisons between the two titration groups were based on 95% CI. The cumulative number of confirmed and/or severe hypoglycaemic events per patient was provided by titration approach and described through descriptive statistics for continuous variables. Moreover, the cumulative mean functions of anytime (24 hours) or nocturnal confirmed [≤70 mg/dL (≤3.9 mmol/L)] and/or severe hypoglycaemia were estimated and graphically presented.

FPG, fasting SMPG and body weight were analysed using the same LMEM described above using the baseline value as a covariate. The frequency and percentage of patients at different HbA_1c_ targets were summarized descriptively by titration group, and chi‐square tests were applied for comparison.

Changes in PAID‐5 and DES‐SF total scores were computed and analysed with a LMEM using the effect of the titration approach and the baseline total score as covariates. DTSQ at the end of the treatment is reported through descriptive statistics in each study arm. Safety endpoints were analysed descriptively.

## 3 RESULTS

### 3.1 Participant flow

Of the 458 patients enrolled, 359 were randomized after exclusion of 72 patients who did not meet the HbA_1c_ criteria (Figure S1). Three hundred and thirty nine (94.4%) patients completed the trial at 46 Italian sites. During the 24‐week study period, 8.6% and 9.4% of patients in the patient and physician groups, respectively, initiated at least one rescue therapy (mainly dipeptidyl peptidase‐4 inhibitors or sodium‐glucose co‐transporter‐2 inhibitors) for poor glycaemic control or post‐prandial hyperglycaemia.

### 3.2 Baseline demographics

Baseline demographics are presented in Table 1 and were not different between the two groups. Over 90% of participants were receiving metformin as monotherapy or combination treatment (Table S2). A complete description of the study population and previous medications has been previously reported.^15^

**Table 1:** Baseline and disease characteristics of the population (ITT population)

Parameter	Total N = 355	Patient‐managed N = 175	Physician‐managed N = 180
Age, years	64.0 (9.8)	64.2 (9.8)	63.7 (9.9)
Age category, %			
18‐64 years	44.8	43.4	46.1
65‐74 years	42.0	43.4	40.6
75‐84 years	13.2	13.1	13.3
Sex (male), %	62.0	62.9	61.1
Diabetes duration, years	11.6 (7.6)	11.6 (7.4)	11.5 (7.8)
HbA_1c_, %	8.8 (0.6)	8.8 (0.7)	8.8 (0.6)
HbA_1c_, mmol/mol	68.2 (7.1)	68.2 (7.3)	68.2 (7.0)
FPG, mg/dL	171 (42)	168 (39)	174 (46)
Median SMPG, mg/dL	152 (range 67‐276)	153 (range 68‐253)	151 (range 67‐276)
BMI, kg/m^2^	30.32 (5.6)	30.54 (6.2)	30.11 (5.0)
eGFR, mL/min/1.73 m^2^ a	85.46 (19.4)	86.41 (18.1)	84.54 (20.7)
eGFR category, n (%)^a^			
<30 mL/min/1.73 m^2^	4 (1.2)	2 (1.2)	2 (1.1)
≥30 to <60 mL/min/1.73 m^2^	38 (10.9)	14 (8.1)	24 (13.6)
≥60 to <90 mL/min/1.73 m^2^	132 (37.8)	65 (37.6)	67 (38.1)
≥90 mL/min/1.73 m^2^	175 (50.1)	92 (53.2)	83 (47.2)
DTSQ treatment satisfaction score^b^	23.23 (7.43)	23.04 (7.38)	23.41 (7.49)
Cardiovascular risk profile^c^, n (%)			
Hypertension	253 (71.27)	124 (70.86)	129 (71.67)
Hyperlipidaemia	195 (54.93)	93 (53.14)	102 (56.67)
Ischemic cardiomyopathy	27 (7.61)	13 (7.43)	14 (7.78)
Atherosclerosis	21 (5.92)	8 (4.97)	13 (7.22)
Revascularisation	21 (5.92)	8 (4.57)	13 (7.22)
Hyperuricemia or gout	15 (4.23)	6 (3.43)	9 (5.0)
Peripheral ischaemia	8 (2.25)	2 (1.14)	6 (3.33)
History of heart failure	4 (1.13)	1 (0.57)	3 (1.67)
History of Stroke	2 (0.56)	0 (0)	2 (1.11)

*Note*: All values are mean, (SD) unless otherwise specified.

Abbreviations: BMI, body mass index; DTSQ, Diabetes Treatment Satisfaction Questionnaire; eGFR, estimated glomerular filtration rate; FPG, fasting plasma glucose; SD, standard deviation; SMPG, self‐monitored plasma glucose.

a Total N = 349, patient‐managed N = 173, physician‐managed N = 176.

b Treatment satisfaction score was calculated as sum of 6 items (1 and from 4 to 8). Treatment satisfaction score ranges from 0 to 36.

c Cardiac disorders were classified as follows: Hypertension: essential hypertension and hypertension; Ischaemic cardiomyopathy: acute coronary syndrome, acute myocardial infarction, myocardial ischaemia, angina pectoris, coronary artery disease, ischaemic cardiomyopathy; Atherosclerosis: carotid arteriosclerosis, arteriosclerosis; Revascularization: coronary (coronary angioplasty, coronary arterial stent insertion, coronary arterial bypass, coronary revascularization, percutaneous coronary intervention), carotid (carotid endarterectomy), peripheral artery (peripheral artery bypass, peripheral endarterectomy, peripheral revascularization), unspecified (endarterectomy, angioplasty). Medical histories were coded using MedDRA dictionary, version 20.1. Each patient could have more than one occurrence of the same medical condition, but they are only counted once for each condition/row overall.

### 3.3 Change in HbA_1c_

**Figure 1:**
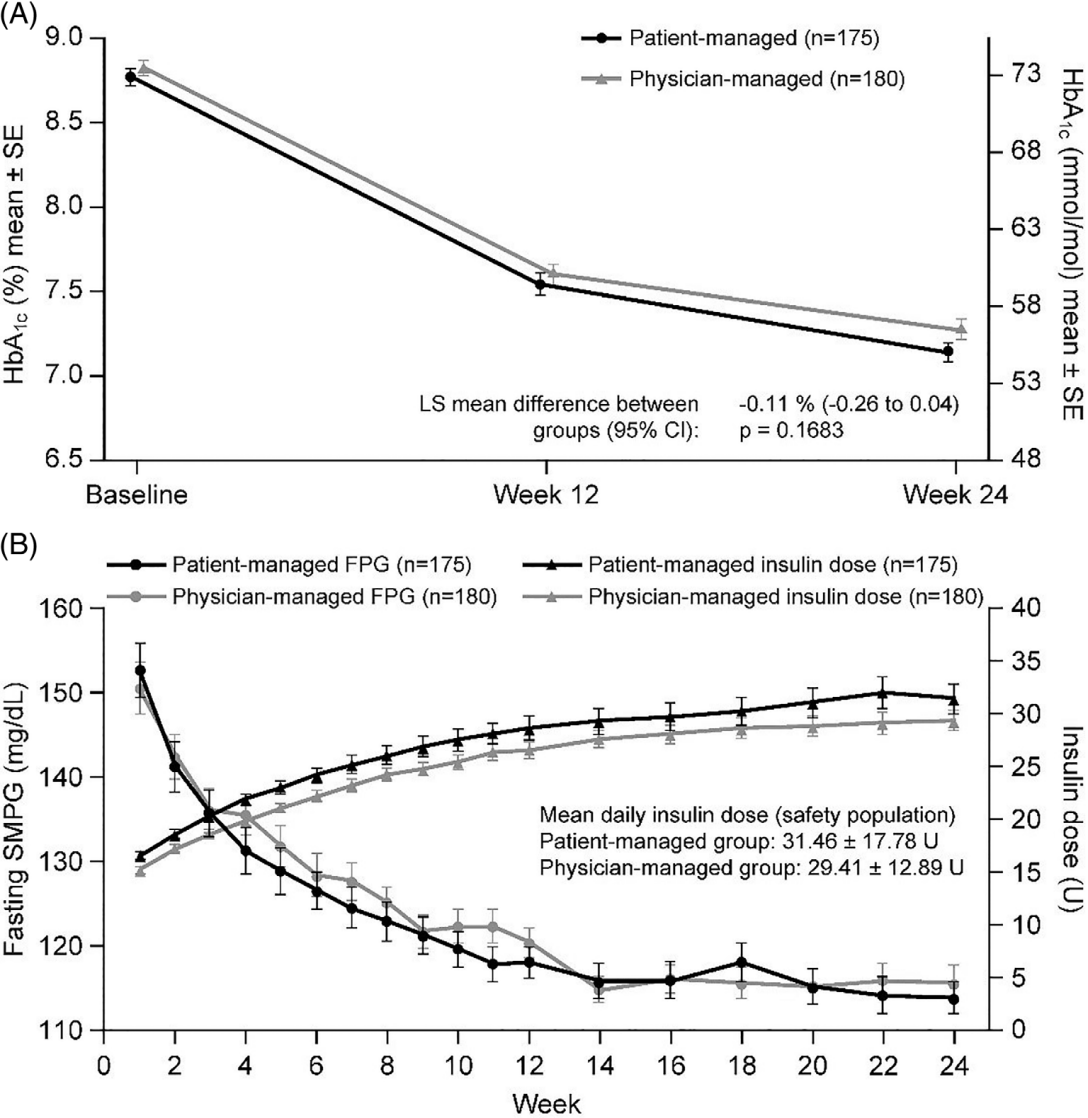
(A) HbA_1c_ reduction between baseline and Week 24 and (B) fasting SMPG over time according to treatment (ITT population). All values are displayed as mean ± SE. CI, confidence interval; FPG, fasting plasma glucose; ITT, intention‐to‐treat; LS, least squared; SE, standard error; SMPG, self‐monitored plasma glucose

At baseline, the mean HbA_1c_ was 8.77% (SD: 0.67) [72.33 mmol/mol (SD: 7.32)] and 8.82% (SD: 0.64) [72.88 (SD: 7.00) mmol/mol] in the patient‐ and physician‐managed titration arms, respectively. For the primary endpoint, the HbA_1c_ reduction from baseline to Week 24 was non‐inferior in the patient‐ vs physician‐managed group. The least squares mean (LSM) change in HbA_1c_ was −1.60% (SE: 0.06) [−17.49 mmol/mol (0.66)] vs −1.49% (SE: 0.06) [−16.29 mmol/mol (SE: 0.66)] in the patient‐ and physician‐managed arms, respectively; LSM difference: −0.11% (95% CI: −0.26 to 0.04) [−1.20 mmol/mol (95% CI: −2.84 to 0.44)] (*P* = .1683) in the ITT population (Figure 1A). The robustness of the primary endpoint was confirmed, as a similar difference was observed between titration groups in the PP population [LS mean difference: −0.14% (−0.30 to 0.01) [−1.53 mmol/mol (−3.28 to 0.11)]], the 95% upper confidence limit of between‐treatment difference being equal to 0.01%, within the non‐inferiority margin of 0.3% (Figure S2). The proportion of participants achieving HbA_1c_ <7.0% was similar in the patient‐managed (40.7%) and physician‐managed (35.2%) groups at Week 24 (*P* = .30).

### 3.4 FPG, fasting SMPG, and insulin dose

**Figure 2:**
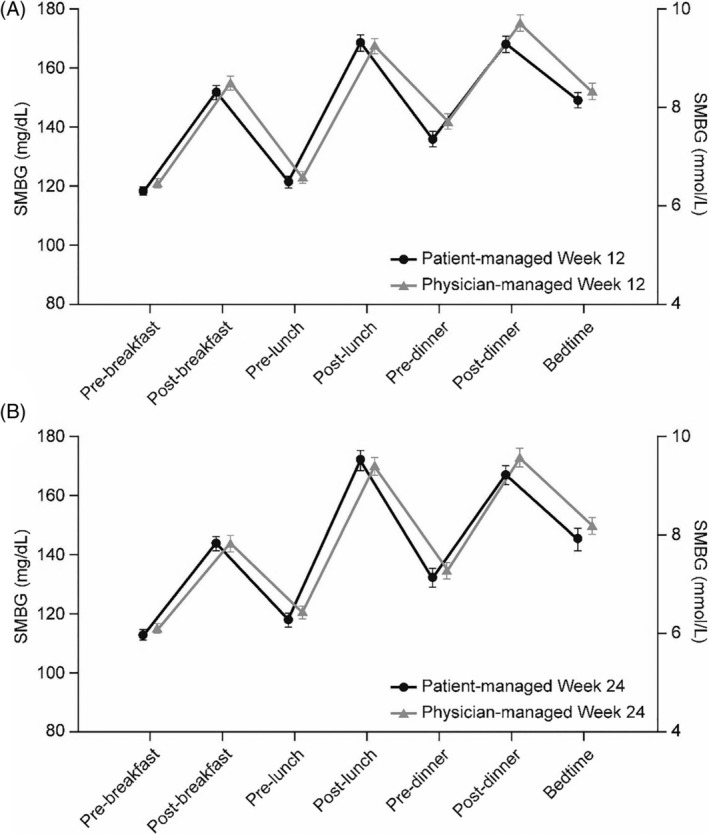
Seven‐point SMPG at (A) Week 12 and (B) Week 24 (ITT population). All values are displayed as mean ± SE. ITT, intention‐to‐treat; SE, standard error; SMPG, self‐monitored plasma glucose

There was no difference in FPG reduction between the patient‐ and physician‐managed groups at Week 24 [LSM difference: −0.05 mg/dL (95% CI: −5.89 to 5.80); *P* = .987]. Mean FPG at 24 weeks was 109 ± 29 mg/dL and 109 ± 28 mg/dL in the patient‐ and physician‐managed groups, respectively. There was no difference in the profiles of progressive reduction in fasting SMPG in the patient‐ and physician‐managed groups [estimated difference between‐groups: −4.25 mg/dL (95% CI: −10.04 to 1.54); *P* = .1499] (Figure 1B). The seven‐point SMPG profiles at Week 12 and Week 24 were similar between the two treatment groups (Figure 2A,B). The insulin dose increased slightly more in the patient‐ vs physician‐managed titration arm over the 24‐week treatment period: from 0.19 ± 0.03 U/kg (15.70 ± 4.19 U) to 0.38 ± 0.17 U/kg (31.46 ± 17.78 U) and from 0.19 ± 0.03 U/kg (15.63 ± 3.75 U) to 0.35 ± 0.15 U/kg (29.41 ± 12.89 U), respectively [LSM difference: 2.43 (95% CI −0.28 to 5.14), *P* = .079] (Figure 1B). During the 24‐week on‐treatment period, a total of 2126 and 1679 dose adjustments were made in the patient‐ and physician‐managed groups, respectively.

### 3.5 Hypoglycaemia

#### 3.5.1 Incidence and rates of confirmed [≤70 mg/dL (≤3.9 mmol/L) or <54 mg/dL (<3.0 mmol/L)] and/or severe nocturnal (00:00‐05:59 hours) hypoglycaemia

**Figure 3:**
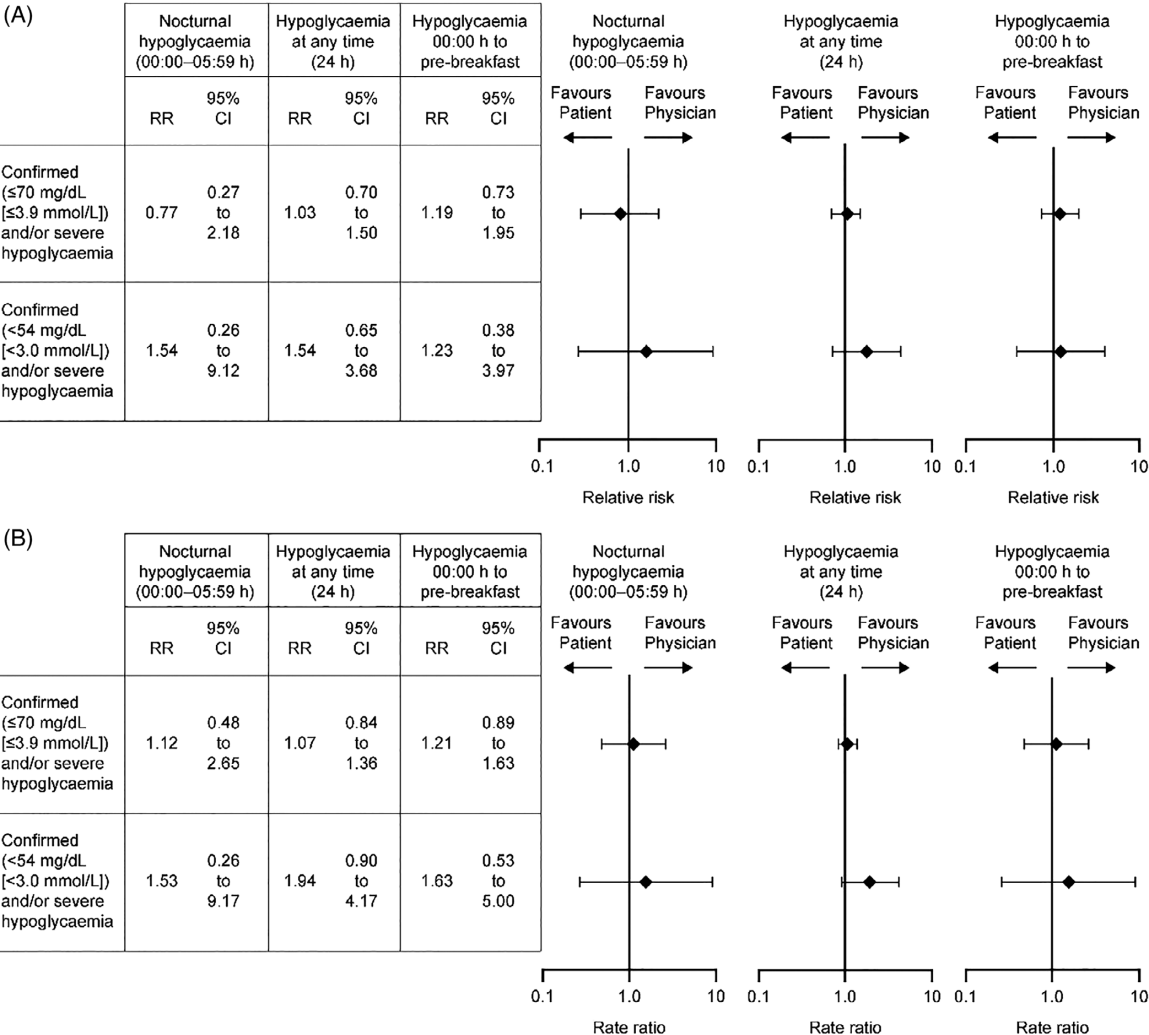
(A) Incidence of participants having ≥1 hypoglycaemic event and (B) rate of hypoglycaemic events (ITT population). CI, confidence interval; ITT, intention‐to‐treat; RR, relative risk in Figure (A) and rate ratio in Figure (B)

In the ITT population, there were no differences between the two titration arms in the cumulative incidence of participants with ≥1 confirmed [≤70 mg/dL (≤3.9 mmol/L)] and/or severe nocturnal (00:00‐05:59 hours) event [3.43% vs 4.44% in the patient‐ vs physician‐managed groups, respectively; relative risk (RR) 0.77 (95% CI: 0.27 to 2.18)] **(**Figure 3A and Table S3). Annualized rates of confirmed [≤70 mg/dL (≤3.9 mmol/L)] and/or severe nocturnal (00:00‐05:59 hours) hypoglycaemia were also not different between the titration groups [11 vs 10 events; 0.13 vs 0.11 events per patient‐year in the patient‐ vs physician‐managed groups; rate ratio: 1.12 (95% CI: 0.48 to 2.65)] (Figure 3B and Table S3). Incidence and rates of confirmed [<54 mg/dL (<3.0 mmol/L)] and/or severe hypoglycaemic events occurring at night (00:00‐05:59 hours) were not different between treatment groups (Figure 3A,B and Table S3).

#### 3.5.2 Incidence and rates of hypoglycaemia at any time of day (24 hours)

No differences were seen in the incidence or annualized rates of confirmed [≤70 mg/dL (≤3.9 mmol/L)] hypoglycaemic events at any time of day (24 hours) between the patient‐ and the physician‐managed groups (Figure 3A,B and Table S3). Incidence and rates of confirmed [<54 mg/dL (<3.0 mmol/L)] and/or severe hypoglycaemic events occurring at any time were not different between treatment groups (Figure 3A,B and Table S3).

#### 3.5.3 Cumulative number of confirmed and/or severe hypoglycaemic events

**Figure 4:**
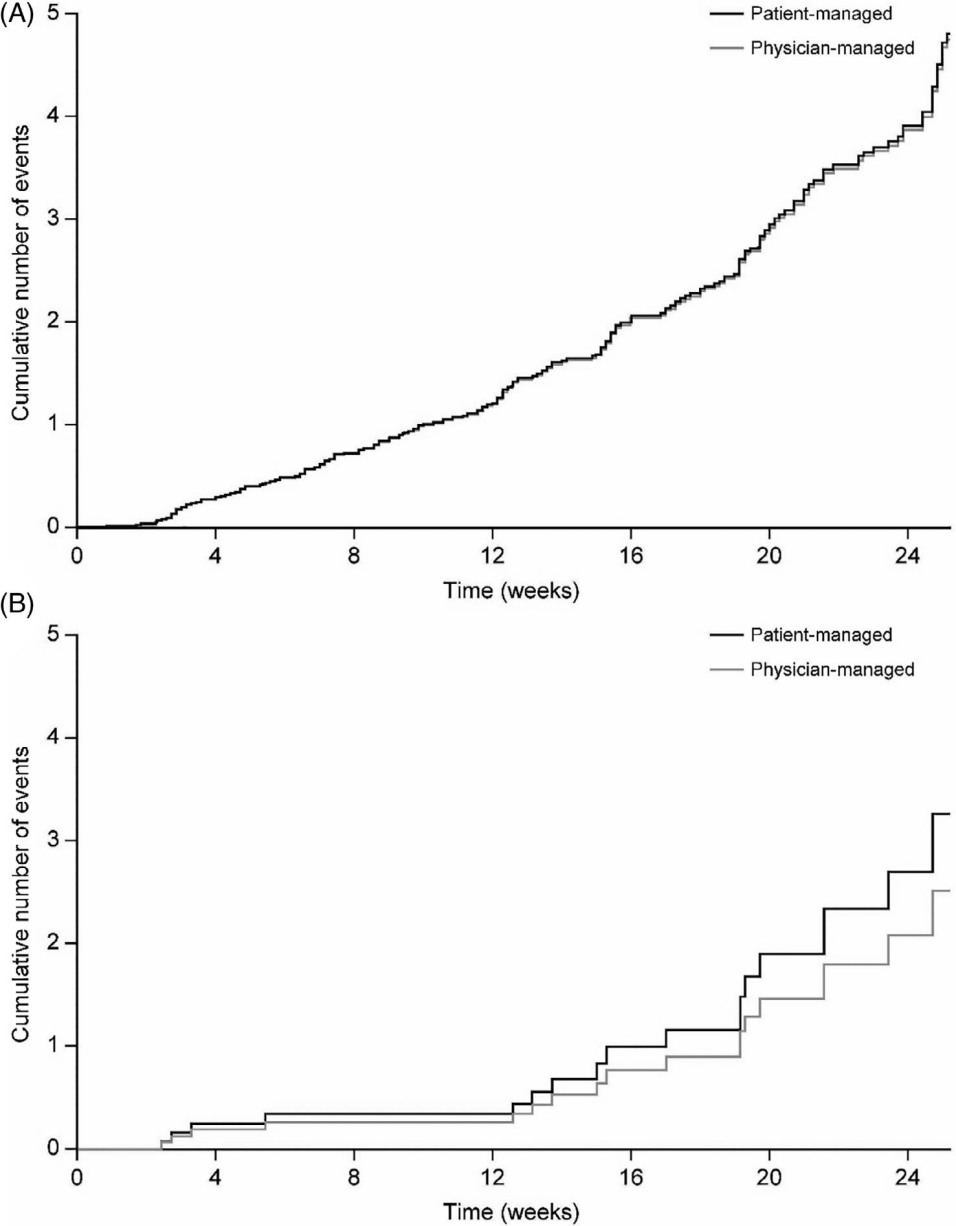
Cumulative mean number of (A) anytime (24 hours) or (B) nocturnal (00:00‐05:59 hours) confirmed and/or severe hypoglycaemic events (ITT population). ITT, intention‐to‐treat

The estimated cumulative mean function of anytime (24 hours) or nocturnal confirmed [≤70 mg/dL (≤3.9 mmol/L)] and/or severe hypoglycaemia was not different between the two treatment groups (Figure 4A,B).

#### 3.5.4 Hypoglycaemia in an expanded nocturnal window

In both groups, most hypoglycaemic events were detected between 06:00 and 09:59 hours (Figure S3). Patient‐ vs physician‐managed titration was associated with numerically fewer hypoglycaemic events between 02:00 and 07:59 hours and between 16:00 and 19:59 hours, but numerically more hypoglycaemic events between 08:00 and 9:59 hours and 20:00 and 21:59 hours (Figure S3). Incidence and rates of confirmed [≤70 mg/dL (≤3.9 mmol/L); <54 mg/dL (<3.0 mmol/L)] and/or severe hypoglycaemic events occurring between 00:00 hour and pre‐breakfast were not different between treatment groups (Figure 3A,B and Table S3).

### 3.6 Severe hypoglycaemia

There were four episodes of severe hypoglycaemia in three patients: one in the patient‐managed group and three in the physician‐managed groups.

### 3.7 Body weight

Body weight remained stable over the 24‐week treatment period, with no changes between baseline and week 24 in the patient‐ and physician‐managed groups [LSM changes (SE): −0.34 (0.31) kg vs 0.10 (0.30) kg, respectively; estimated difference between groups: −0.43 kg (95% CI: −1.21 to 0.34); *P* = .30].

### 3.8 Composite endpoints

The proportion of patients achieving HbA_1c_ <7.0% without severe and/or confirmed hypoglycaemia were also not different between the groups (29.3% and 26.1% in the patient‐ and physician‐managed groups, respectively) (*P* = .50). There was also no significant difference between the patient‐ and physician‐managed groups among patients who achieved HbA_1c_ <7.0% without severe and/or confirmed hypoglycaemic events and no weight increase (19.8% vs 14.6%, respectively) (*P* = .21).

### 3.9 Patient‐reported outcomes

There was no difference between study groups for improvement in DTSQ treatment satisfaction between baseline and Week 24 (Figure S4). For the DES‐SF questionnaire, the total LS mean scores (SE) increased (improved) by 0.21 (0.05) points in the patient‐ and 0.19 (0.04) points in the physician‐managed groups [estimated difference between groups: 0.02 [95% CI: −0.09 to 0.13]; *P* = .70]. Total LSM scores (SE) in the PAID‐5 questionnaire were reduced (improved) by −1.32 (0.42) points in the patient‐managed group and −1.51 (0.41) points in the physician‐managed group [estimated difference between groups: 0.19 (95% CI: −0.81 to 1.18); *P* = .7093].

### 3.10 Adverse events

A summary of treatment‐emergent adverse events (TEAEs) can be found in Table S4. There were no drug‐related serious adverse events. Three TEAEs [bilateral peripheral oedema (n = 1) in the patient‐managed group and coronary artery disease (n = 1) and pregnancy (n = 1) in the physician‐managed group] led to treatment discontinuation. Seven TEAEs were suspected of being treatment‐related (five in the patient‐ and two in the physician‐managed group) and were as follows: mild oedema, severe hypoglycaemia, moderate weight increase, injection site mild pain, and injection site mild pruritus in the patient‐managed group and injection site mild pain and injection site mild pruritus in the physician‐managed group.

## 4 DISCUSSION

ITAS is the first randomized controlled trial specifically comparing the efficacy and safety of initiation and titration of Gla‐300 managed by either the patient (nurse‐assisted) or physician, in an insulin‐naïve population with uncontrolled T2DM, after withdrawal of SU/glinides. The results of ITAS show that patient‐managed titration of Gla‐300 achieves non‐inferior glucose control with similarly low risk of hypoglycaemia compared with physician‐managed titration, with a successful end‐of‐study HbA_1c_ close to 7.0%. Taken together, the results of ITAS demonstrate not only the efficacy of Gla‐300 using a simpler patient‐ vs a physician‐managed titration process but also the safety of such an approach. Of note, the results of ITAS were obtained in the context of identical numbers of visits/contacts in the two arms of the study, that is, with nurses and physicians, respectively. These positive results of ITAS using Gla‐300 may help in reducing the barriers to initiation and titration of BI in uncontrolled T2DM people who need to initiate BI. Several previous studies have compared patient‐ and physician‐managed titration using BI Gla‐100 [AT.LANTUS (insulin‐naïve and pre‐treated participants)^8^ and ATLAS (insulin‐naïve participants)^9^]. These two studies showed even greater reductions in HbA_1c_ with patient‐ vs physician‐managed titration. However, overall incidence of hypoglycaemia was higher in the patient‐ vs physician‐managed titration arms in AT.LANTUS (*P* < .01) and ATLAS (*P* = .02 for symptomatic hypoglycaemia; *P* = .002 for nocturnal hypoglycaemia). In ITAS, there is a lower risk of hypoglycaemia (any definition) in both patient‐ and physician‐managed BI titration groups, likely because the insulin‐naïve population studied had lower hypoglycaemia risk, and also because Gla‐300 carries a lower risk of hypoglycaemia vs Gla‐100.^13^

Recently, the TAKE CONTROL study^14^ investigated self‐titration of Gla‐300 by patients vs physicians and reported non‐inferiority and superiority of patient‐titration with regards to HbA_1c_ reduction. However, this study was made in a population heterogeneous for antecedent insulin use since the majority of subjects at baseline were on long‐term insulin and therefore familiar with this treatment. Incidence and rates of hypoglycaemia were higher in TAKE CONTROL compared to ITAS, likely because of the greater hypoglycaemia risk (longer diabetes duration, antecedent insulin treatment in >60% of subjects, continued use of SU/glinides in TAKE CONTROL). Although continuation vs withdrawal of SU at time of BI initiation is still a debated question, evidence indicates that continuation of SU upon initiating BI increases the risk for hypoglycaemia,^16^ possibly confounding the interpretation of the risk specifically due to BI. The withdrawal of SU is also aligned with the Italian Standards for the Treatment of Diabetes Mellitus, which discourages use of SU/glinides upon initiation of BI.^18^ Finally, the titration algorithm appears smoother and safer in ITAS vs that of TAKE CONTROL, with smaller insulin dose changes targeting safer glycaemic targets.^13^

Interestingly, in ITAS, there is a lower risk of hypoglycaemia also when the comparison is done vs another study, EDITION 3, in which BI was initiated in insulin‐naïve subjects after withdrawal of SU/glinides.^19^ One should note that the method of collection of hypoglycaemia events in ITAS (use of patients' diaries and e‐CRF) might have led to an underestimation of its real occurrence compared to a more frequent determination of SMPG prior to each visit, as in EDITION 3.^19^ However, this was similar to TAKE CONTROL,^14^ suggesting that the low hypoglycaemia risk in ITAS may be explained also with successful education of patients and dedication of physicians and nurses to titration of BI. The intensity of the educational program in ITAS is indicated by the higher number of insulin dose changes in the patient (nurse‐assisted) vs physician group. In this context, ITAS findings may indicate the need for an ad hoc intervention in the local model of care aiming at establishing a specialized nurse devoted to patient support in BI initiation and titration, lightening physician tasks, and improving patient access, motivation, and adherence.

Regardless of the mechanism, reducing hypoglycaemia on BI treatment in T2DM is an important goal to improve long‐term adherence of patients to insulin treatment and titration to target, as demonstrated by the high retention of patients in ITAS in both groups for the entire duration of the study. Minimizing the risk of even non‐severe hypoglycaemia, as in ITAS, is important to prevent long‐term hypoglycaemia unawareness,^20^ and the subsequent risk for severe hypoglycaemia,^21^ which is associated with higher risk of cardiovascular morbidity and mortality.^22^, ^23^

Interestingly, there was no increase in body weight with Gla‐300 neither in patient‐ nor in physician‐managed BI titration, in line with previous observations.^13^

As in TAKE CONTROL, ITAS reported no difference in total PRO scores in the patient‐ and physician‐managed titration arms, demonstrating that patients felt comfortable with self‐titration. In addition, incidence of TEAEs was not different in the patient‐ and physician‐managed groups, as shown in TAKE CONTROL, AT.LANTUS, and ATLAS.^8^, ^9^, ^14^

ITAS tried to mimic certain features of pragmatism (ie, not too selected study population; easy and safe titration algorithm) comparing physician managed titration with patient (nurse‐assisted) self‐titration. Both approaches are similarly successful and safe in ITAS, thus proving the feasibility and convenience of the simpler patient (nurse‐assisted) approach.

The limitations of this study include the open‐label design. It should also be noted that the good results of ITAS have been obtained with a number of visits/contacts, which do not necessarily reflect the lower level of interaction that patients would often receive in routine clinical practice in diabetes clinics. Finally, hypoglycaemia events were pragmatically reported and analysed by patient diaries or e‐CRF and not by independent glucose monitoring tools.

In conclusion, ITAS provides additional evidence that initiation and titration of BI Gla‐300 are not only efficacious as several previous studies have shown but also safe with low risk for any hypoglycaemia, especially when SU/glinides, a likely confounder, are suspended. Interestingly, these positive results, which include neutrality of BI Gla‐300 on body weight, are achieved similarly when either patients (nurse assisted) or physicians manage BI titration. This encourages development of programmes for wider use of the patient‐managed (nurse‐assisted) approach for BI titration, shifting the locus of control to fight clinical inertia.

## CONFLICT OF INTEREST

R.C.B. has received honoraria or consulting fees from Sanofi, Merck Sharp & Dohme, Eli Lilly, Johnson & Johnson, Bristol‐Myers Squibb, AstraZeneca and Janssen. A.G. has received honoraria or consulting fees from Amgen, AstraZeneca, Boehringer Ingelheim, Eli Lilly, MSD, Mundipharma, Sanofi and research funding from AstraZeneca. R.B. has received honoraria or consulting fees from Sanofi, Eli Lilly, Abbott and Novo Nordisk. G.P. has received honoraria or consulting fees from Novo Nordisk, Sanofi, Eli Lilly, AstraZeneca, Boehringer Ingelheim, Takeda, Abbott, Janssen and Merck Sharp & Dohme. D.C. has received honoraria or consulting fees from Eli Lilly, Novo Nordisk, Roche Diagnostics and Sanofi. A.A. has received honoraria or consulting fees from Novo Nordisk, Sanofi, Eli Lilly, AstraZeneca, Boehringer Ingelheim, Takeda and Servier and research funding from AstraZeneca and Vifor Pharma. G.A. has received honoraria or consulting fees from Sanofi, AstraZeneca, IBSA Institut Biochimique and Novartis and research funding from Sanofi, AstraZeneca and Novo Nordisk. M.L. is an employee of Sanofi and holds stocks/shares in Sanofi. C.F. has received honoraria or consulting fees from Sanofi and non‐financial support from Menarini. G.B.B. has received honoraria or consulting fees from Sanofi and Menarini and research funding and speakers' bureau fees from Sanofi.

## AUTHORS' CONTRIBUTION

All authors have read and approved the final manuscript. R.C.B. participated as an investigator, provided comments and input to all drafts of the manuscript and interpretation of the results. A.G. participated as an investigator, provided comments and input to all drafts of the manuscript and interpretation of the results. R.B. participated as an investigator, provided comments and input to all drafts of the manuscript and interpretation of the results. G.P. participated as an investigator, provided comments and input to all drafts of the manuscript and interpretation of the results. D.C. participated as an investigator, provided comments and input to all drafts of the manuscript and interpretation of the results. A.A. participated as an investigator, provided comments and input to all drafts of the manuscript and interpretation of the results. G.A. participated as an investigator, provided comments and input to all drafts of the manuscript and interpretation of the results. M.L. provided comments and input to all drafts of the manuscript and interpretation of the results. C.F. participated as an investigator, provided comments and input to all drafts of the manuscript and interpretation of the results. G.B.B. participated as an investigator, provided comments and input to all drafts of the manuscript and interpretation of the results.

## Supporting information


**Table S1** Titration algorithm used by patients and physicians.
**Table S2**. Glucose lowering medications at baseline (ITT population)
**Table S3.** Relative risk and annualized rate of nocturnal, 00:00 hour to pre‐breakfast and anytime (24 hours) hypoglycaemia from baseline to Week 24 (ITT population)
**Table S4.** Summary of patients with treatment‐emergent adverse events
**Figure S1.** Study flow
**Figure S2.** HbA_1c_ reduction between baseline and Week 24 (PP population)
**Figure S3.** Hypoglycaemia in an expanded nocturnal window or at any time of day (ITT population)
**Figure S4.** DTSQ at baseline and Week 24 (ITT population)
