# Supplementary material for: Comparable efficacy with similarly low risk of hypoglycaemia in patient‐ vs physician‐managed basal insulin initiation and titration in insulin‐naïve type 2 diabetic subjects: The Italian Titration Approach Study
Source: Diabetes Metab Res Rev. 2020 Apr 5;36(6):e3304. doi: 10.1002/dmrr.3304 (PMC7540052; doi:10.1002/dmrr.3304)
Supplement: Supplementary file 1 — Table S1 Titration algorithm used by patients and physicians. Table S2. Glucose lowering medications at baseline (ITT population) Table S3. Relative risk and annualized rate of nocturnal, 00:00 hour to pre‐breakfast and anytime (24 hours) hypoglycaemia from baseline to Week 24 (ITT population) Table S4. Summary of patients with treatment‐emergent adverse events Figure S1. Study flow Figure S2. HbA1c reduction between baseline and Week 24 (PP population) Figure S3. Hypoglycaemia in an expanded nocturnal window or at any time of day (ITT population) Figure S4. DTSQ at baseline and Week 24 (ITT population) [file DMRR-36-e3304-s001.docx]

# Supplementary appendix

## Supplementary tables

**Supplementary table 1.** Titration algorithm used by patients and physicians.

|  | **Gla-300 dose adjustment (Units)^†^** | |
| --- | --- | --- |
| Fasting SMPG* | **Patient-managed** | **Physician-managed** |
| **>180 mg/dL  (>10 mmol/L)** | +4 | |
| **110–180 mg/dL  (6.1–10 mmol/L)** | +2 | |
| **80–110 mg/dL  (4.4–6.1 mmol/L)** | No change | |
| **<80 mg/dL  (<4.4 mmol/L)** | -2 | |
| **<54 mg/dL or occurrence of ≥2 symptomatic or 1 severe episode of hypoglycaemia in the preceding week** | Contact physician | At physician’s discretion |

^†^Doses in the physician-managed titration-arm were adjusted at each visit or telephone contact (weekly until week 12 and then bi-weekly until week 24) and not more often than every 3–4 days in the self-managed titration arm, to achieve a target range for fasting SMPG of 80-110 mg/dL.

*Fasting SMPG values measured on 3 consecutive days, of which the last is the day when titration is to occur.

**Supplementary table 2**. Glucose lowering medications at baseline (ITT population)

|  | **Total** | | **Patient-managed** | | **Physician-managed** | |
| --- | --- | --- | --- | --- | --- | --- |
|  | **N=355** | | **N=175** | | **N=180** | |
|  | **n** | **%** | **n** | **%** | **n** | **%** |
| **Number of glucose lowering medications at baseline** |  |  |  |  |  |  |
| 0 | 5 | 1.41 | 4 | 2.29 | 1 | 0.56 |
| 1 | 106 | 29.86 | 50 | 28.57 | 56 | 31.11 |
| 2 | 169 | 47.61 | 80 | 45.71 | 89 | 49.44 |
| 3 | 71 | 20.00 | 39 | 22.29 | 32 | 17.78 |
| 4 | 4 | 1.13 | 2 | 1.14 | 2 | 1.11 |
| **Drug class** |  |  |  |  |  |  |
| Metformin | 327 | 92.11 | 160 | 91.43 | 167 | 92.78 |
| Dipeptidyl peptidase-4 inhibitors | 114 | 32.11 | 61 | 34.86 | 53 | 29.44 |
| Sulphonylureas | 99 | 27.89 | 50 | 28.57 | 49 | 27.22 |
| Sodium-glucose  co-transporter 2 inhibitors | 38 | 10.70 | 16 | 9.14 | 22 | 12.22 |
| Glucagon-like peptide-1 receptor agonist | 31 | 8.73 | 17 | 9.71 | 14 | 7.78 |
| Repaglinide | 30 | 8.45 | 12 | 6.86 | 18 | 10.00 |
| Pioglitazone | 23 | 6.48 | 12 | 6.86 | 11 | 6.11 |
| Acarbose | 11 | 3.10 | 7 | 4.00 | 4 | 2.22 |

ITT, intention-to-treat

**Supplementary table 3.** Relative risk and annualized rate of nocturnal, 00:00 h to pre-breakfast and anytime (24 h) hypoglycaemia from baseline to Week 24 (ITT population)

| **Period** | **Relative risk of** ≥1 **confirmed hypoglycaemic event** | | | | | |
| --- | --- | --- | --- | --- | --- | --- |
|  | **HYPOGLYCAEMIA INCIDENCE** | **PATIENT-MANAGED**  **(N=175)** | | **PHYSICIAN-MANAGED**  **(N=180)** | | **RELATIVE RISK**  **[95% CI]** |
|  |  | **Patients with event** | **%**  **patients** | **Patients with event** | **%**  **patients** |  |
| **Nocturnal (00:00–05:59 h)^†^** | **Patients with ≥1 confirmed (≤70 mg/dL) and/or severe hypoglycaemic episodes** | **6** | **3.43** | **8** | **4.44** | **0.77 (0.27 to 2.18)** |
| 00:00 to pre-breakfast |  | 29 | 16.57 | 25 | 13.89 | 1.19 (0.73 to 1.95) |
| Anytime (24 h) |  | 41 | 23.43 | 41 | 22.78 | 1.03 (0.70 to 1.50) |
| Nocturnal (00:00–05:59 h) | Patients with ≥1 confirmed (<54 mg/dL) and/or severe hypoglycaemic episodes | 3 | 1.71 | 2 | 1.11 | 1.54 (0.26 to 9.12) |
| 00:00 to pre-breakfast |  | 6 | 3.43 | 5 | 2.78 | 1.23 (0.38 to 3.97) |
| Anytime (24 h) |  | 12 | 6.86 | 7 | 4.44 | 1.76 (0.71 to 4.37) |
|  | **Ratio of the annualized hypoglycaemic event rate** | | | | | |
| **Period** | **NUMBER OF HYPOGLYCAEMIC EPISODES** | **PATIENT-MANAGED**  **(N=194)** | | **PHYSICIAN-MANAGED**  **(N=149)** | | **RATE RATIO (95% CI)** |
|  |  | **Number of  events** | **Rate (95% CI)** | **Number of events** | **Rate [95% CI]** |  |
| Nocturnal (00:00–05:59 h) | Number of confirmed (≤70 mg/dL) and/or severe hypoglycaemic episodes | 11 | 0.13 (0.05 to 0.20) | 10 | 0.11 (0.04 to 0.18) | 1.12 (0.48 to 2.65) |
| 00:00–pre-breakfast |  | 92 | 1.07 (0.85 to 1.29) | 78 | 0.89 (0.69 to 1.08) | 1.21 (0.89 to 1.63) |
| Anytime (24 h) |  | 133 | 1.55 (1.28 to 1.81) | 127 | 1.44 (1.19 to 1.70) | 1.07 (0.84 to 1.36) |
| Nocturnal (00:00–05:59 h) | Number of confirmed (<54 mg/dL) and/or severe hypoglycaemic episodes | 3 | 0.03 (0.00 to 0.07) | 2 | 0.02 (0.00 to 0.05) | 1.53 (0.26 to 9.17) |
| 00:00-pre-breakfast |  | 8 | 0.09 (0.03 to 0.16) | 5 | 0.06 (0.01 to 0.11) | 1.63 (0.53 to 5.00) |
| Anytime (24 h) |  | 19 | 0.22 (0.12 to 0.32) | 10 | 0.11 (0.04 to 0.18) | 1.94 (0.90 to 4.17) |

^†^Primary endpoint (also shown in bold). CI, confidence interval; ITT, intention-to-treat

**Supplementary table 4.** Summary of patients with treatment-emergent adverse events

|  | **Total** | | **Patient-managed** | | **Physician-managed** | |
| --- | --- | --- | --- | --- | --- | --- |
|  | **N=355** | | **N=175** | | **N=180** | |
|  | **n** | **%** | **n** | **%** | **n** | **%** |
| **Event** |  |  |  |  |  |  |
| Number of TEAEs | 173 | – | 91 | – | 82 | – |
| Patients with TEAEs | 87 | 24.51 | 46 | 26.29 | 41 | 22.78 |
| Patients with serious TEAEs | 14 | 3.94 | 3 | 1.71 | 11 | 6.11 |
| Patients with severe TEAEs | 9 | 2.54 | 3 | 1.71 | 6 | 3.33 |
| Patients with drug-related TEAEs | 5 | 1.41 | 4 | 2.29 | 1 | 0.56 |
| Patients with treatment permanently discontinued owing to TEAEs | 3 | 0.85 | 1 | 0.57 | 2 | 1.11 |
| Patients with TEAEs classified as events of interest | 1 | 0.28 | 0 | 0.00 | 1 | 0.56 |

TEAE, treatment-emergent adverse event

## Supplementary figures

**Supplementary figure 1.** Study flow


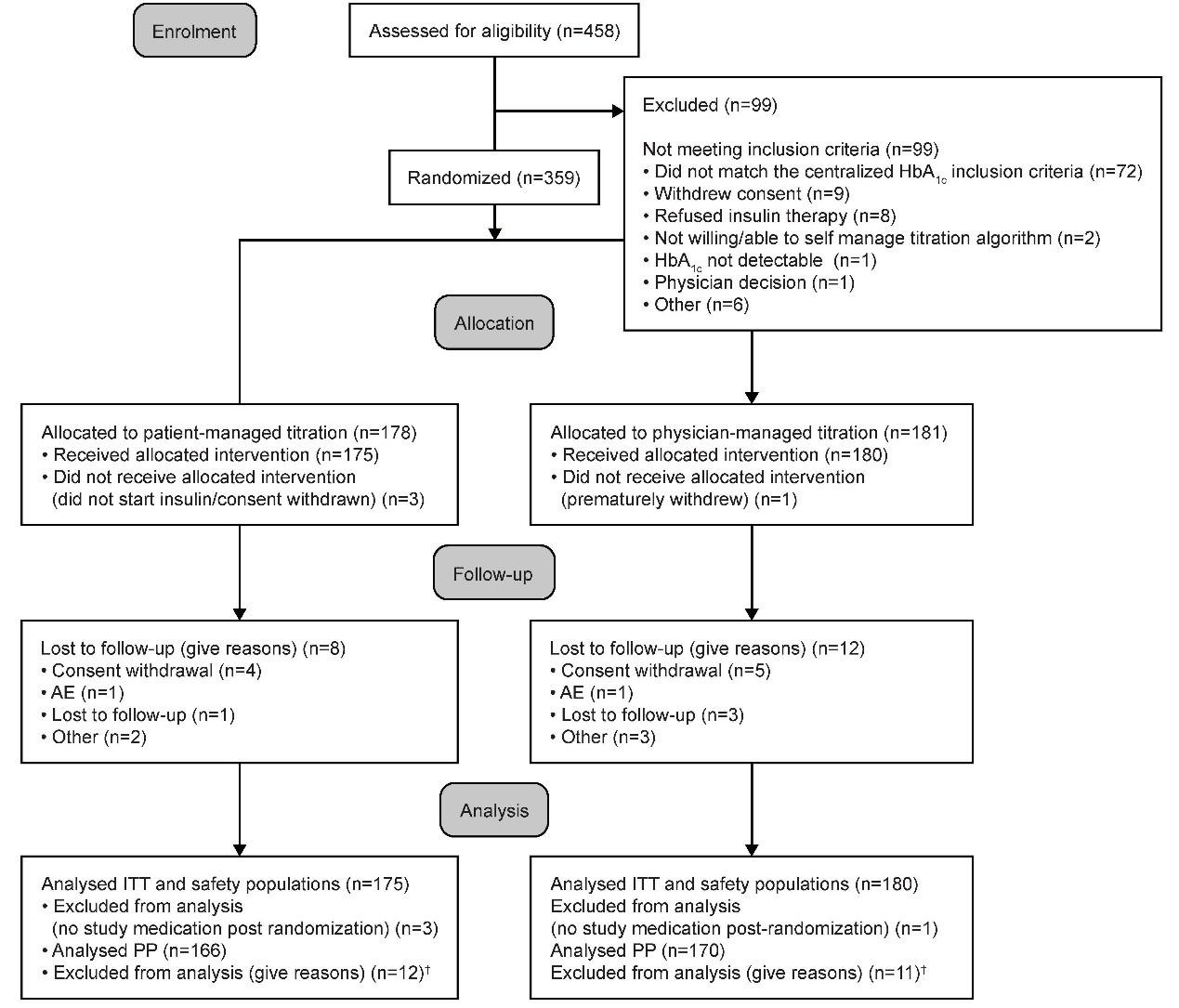


Both the ITT and safety populations included 175 patients in the patient-managed and 180 patients in the physician-managed groups. The PP population included 166 and 170 participants in the patient-managed and physician-managed groups, respectively.

^†^23 participants (6.41%) were excluded from the PP population, the most common reason was not completing at least 12 weeks of treatment (n=15). Other reasons were using a titration approach different from the one assigned, use of sulphonylureas or glinides, not taking any study medication after randomization and violating the inclusion criteria of being insulin-naïve (treated with oral antihyperglycemic drugs); patients not willing to interrupt treatment with sulphonylureas/glinides at randomization.

AE, adverse event; ITT, intention-to-treat; PP, per-protocol

**Supplementary figure 2.** HbA_1c_ reduction between baseline and Week 24 (PP population)

**
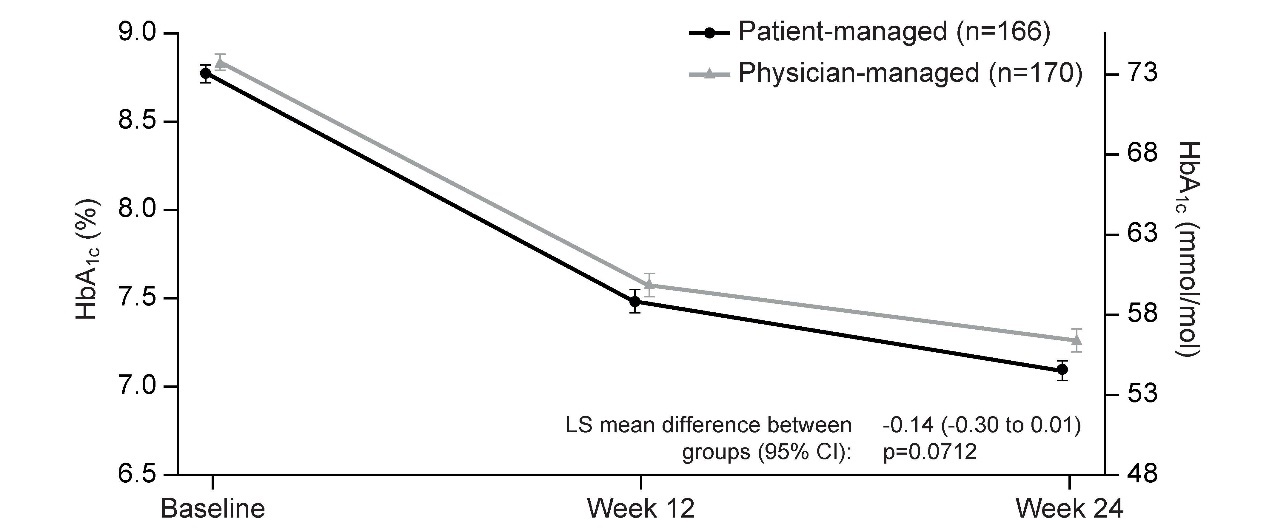
**

All values displayed are mean ± SE

CI, confidence interval; LS, least squares; PP, per-protocol; SE, standard error

**Supplementary figure 3.** Hypoglycaemia in an expanded nocturnal window or at any time of day (ITT population)


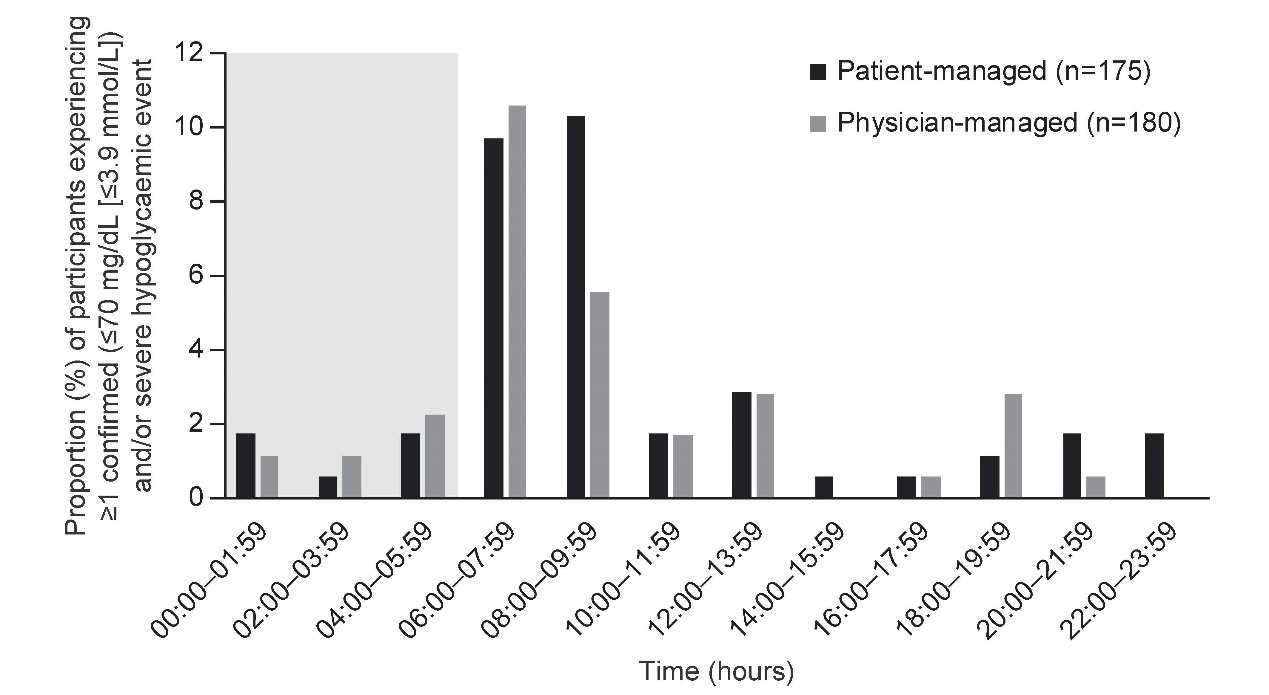


**Supplementary figure 4.** DTSQ at baseline and Week 24 (ITT population)


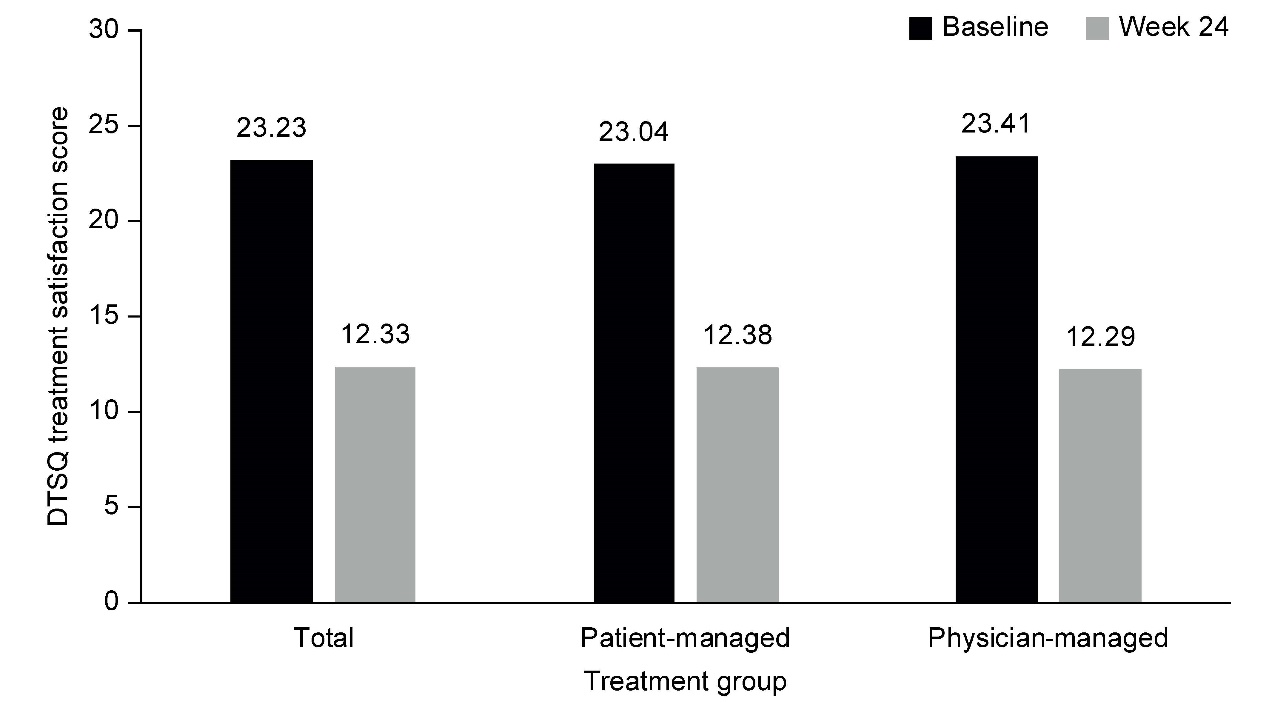


DTSQ, Diabetes Treatment Satisfaction Questionnaire; ITT, intention-to-treat
